# Complete Mitogenomes of Three Carangidae (Perciformes) Fishes: Genome Description and Phylogenetic Considerations

**DOI:** 10.3390/ijms21134685

**Published:** 2020-06-30

**Authors:** Zhenhai Li, Min Li, Shannan Xu, Li Liu, Zuozhi Chen, Keshu Zou

**Affiliations:** 1 Joint Laboratory of Guangdong Province and Hong Kong Region on Marine Bioresource Conservation and Exploitation, College of Marine Science, South China Agriculture University, Guangzhou 510642, China; lzh1097146593@163.com (Z.L.); liuli@scau.edu.cn (L.L.); 2 Key Laboratory of Open-Sea Fishery Development, Ministry of Agriculture and Rural Affairs, South China Sea Fisheries Research Institute, Chinese Academy of Fishery Sciences, Guangzhou 510300, China; limin@scsfri.ac.cn (M.L.); xushannan@scsfri.ac.cn (S.X.); 3 Southern Marine Science and Engineering Guangdong Laboratory (Guangzhou), Guangzhou 511458, China; 4 Guangdong Laboratory for Lingnan Modern Agriculture, South China Agriculture University, Guangzhou 510642, China

**Keywords:** *Alectis indicus*, *Decapterus tabl*, *Alepes djedaba*, comparative characterization, mitochondrial genome, phylogeneny

## Abstract

Carangidae are ecologically and economically important marine fish. The complete mitogenomes of three Carangidae species (*Alectis indicus*, *Decapterus tabl*, and *Alepes djedaba*) were sequenced, characterized, and compared with 29 other species of the family Carangidae in this study. The length of the three mitogenomes ranged from 16,530 to 16,610 bp, and the structures included 2 rRNA genes (*12S rRNA* and *16S rRNA*), 1 control region (a non-coding region), 13 protein-coding genes, and 22 tRNA genes. Among the 22 tRNA genes, only *tRNA-Ser* (GCT) was not folded into a typical cloverleaf secondary structure and had no recognizable DHU stem. The full-length sequences and protein-coding genes (PCGs) of the mitogenomes of the three species all had obvious AT biases. The majority of the AT-skew and GC-skew values of the PCGs among the three species were negative, demonstrating bases T and C were more plentiful than A and G. Analyses of Ka/Ks and overall p-genetic distance demonstrated that *ATP8* showed the highest evolutionary rate and *COXI*/*COXII* were the most conserved genes in the three species. The phylogenetic tree based on PCGs sequences of mitogenomes using maximum likelihood and Bayesian inference analyses showed that three clades were divided corresponding to the subfamilies Caranginae, Naucratinae, and Trachinotinae. The monophyly of each superfamily was generally well supported. The divergence time analyses showed that Carangidae evolved during three geological periods, the Cretaceous, Paleogene, and Neogene. *A. indicus* began to differentiate from other species about 27.20 million years ago (Mya) in the early Miocene, while *D. tabl* (21.25 Mya) and *A. djedaba* (14.67 Mya) differentiated in the middle Oligocene.

## 1. Introduction

The typical mitochondrial genome (mitogenome) of fish consists of circular double-stranded DNA molecules totaling 16~19 kb, which are composed of 37 genes comprising two rRNA genes (*12S* and *16S rDNA*), 22 tRNA genes, 13 protein-coding genes (PCGs), and a non-coding control region that possesses cis regulatory elements [1]. The mitochondrial DNA (mtDNA) sequence has compact gene arrangement, short coalescence time, rapid evolutionary rate, and maternal inheritance, allowing scientists to use them for phylogenetic analyses, population genetics, and taxonomic studies [2]. Currently, mtDNA sequences (both partial and complete) are used extensively as molecular markers for species identification [3,4,5,6,7,8]. However, incomplete mitochondrial gene sequences have limited the ability to identify complex genetic evolutionary relationships in many fish lineages [9].

The Carangidae (Teleostei: Perciformes) are a diverse family of marine fishes that include both ecologically and economically important species [10,11]. The Indian threadfish (*Alectis indicus* Rüppell, 1830), roughear scad (*Decapterus tabl* Berry, 1968), and shrimp scad (*Alepes djedaba* Forsskål, 1775) are three Carangidae fishes that play important roles as fishery resources in the Northwest Pacific [12]. *D. tabl* contribute a lot to the world’s fishery production, and it is the highest-yielding fish in commercial fisheries in some countries such as Japan, the Philippines, and Nigeria [13]. *A. indicus* has a unique shape and is popular in the ornamental fish industry [14]. Recent studies have examined their growth, mortality, age composition, and fisheries yields [15,16,17], but not their genetic characteristics and evolutionary patterns. Furthermore, their phylogenetic positions in Carangidae were also unclear. Although some of the mitochondrial genes [18,19] were used as molecular markers to elucidate phylogenetic relationship in Carangidae, the sequences of these fragments contained limited information and may not fully reflect the genetic relationship among Carangidae species.

In this study, the complete mitogenomes of *A. indicus*, *D. tabl,* and *A. djedaba* were sequenced using next-generation sequencing. The genome structure and composition were analyzed, including AT- and GC-skew, evolutionary rates, pairwise genetic distance, and codon preferences. In addition, the CGView comparison tool (CCT) was also used to make a visualization map that can directly reflect the differences of mitochondrial sequences of the family Carangidae, and some clusters of orthologous groups (COG) were identified. The phylogenetic relationship of the species in Carangidae was reconstructed, and their divergence times were estimated. The sequence data of the three Carangidae could not only provide useful information for species identification, conservation genetics, and molecular phylogenetics but also provide further insight into the deep level evolutionary relationships among these commercially important fishes.

## 2. Results and Discussion

### 2.1. Genome Organization and Base Composition

**Figure 1:**
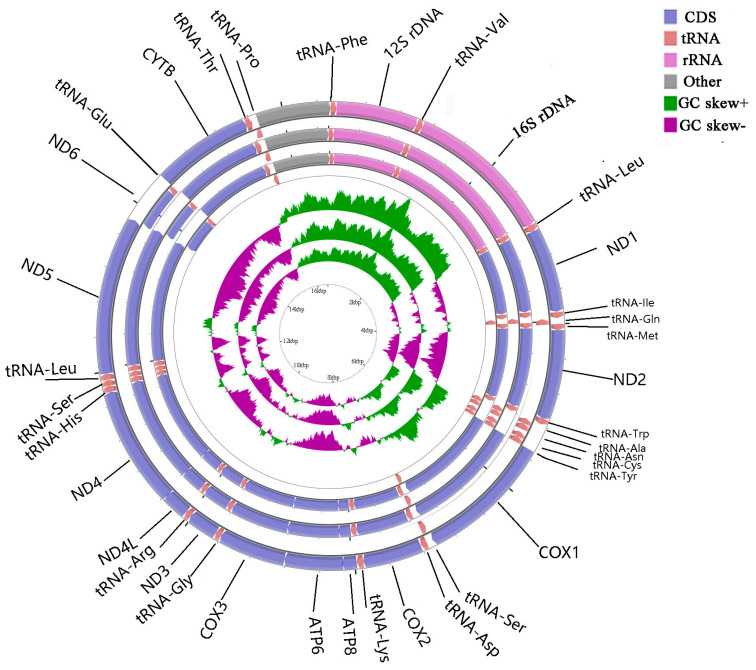
Gene map of the complete mitogenomes of *Alectis indicus*, *Decapterus tabl,* and *Alepes djedaba.* Note: The larger ring indicates the gene arrangement and distribution; the genes on the outer circle are encoded by the H-strand, and those on the inner circle by the L-strand. The smaller ring indicates the GC content. *ND1–6*, *NADH* dehydrogenase subunits 1–6; *COXI–III*, cytochrome c oxidase subunits 1–3; *ATP6* and *ATP8*, ATPase subunits 6 and 8; *Cytb*, cytochrome b.

The complete mitogenomes of *A. indicus*, *D. tabl*, and *A. djedaba* were 16,553, 16,545, and 16,563 bp in length, respectively. Most genes were encoded by the heavy (H) chain, except for eight tRNA genes (Tyr, Cys, Glu, Pro, Ala, Asn, Ser, and Gln) and the *ND6* gene, which were encoded by the light (L) chain (Figure 1).

The GC content of the 37 genes varied from 29.85% to 58.33%, and eleven intergenic spacers (IGSs) were identified totaling 66 bp in all three fishes (Table 1). The largest IGS was located on the L strand between *tRNA-Cys* and *tRNA-Asn*, which was 38, 37, and 37 bp in *A. indicus*, *D. tabl*, and *A. djedaba*, respectively (Table 1). The overlap of mitochondrial genes made the genome even tighter. Seven overlaps were identified in each species, totaling 25 bp in *A. indicus*, 22 bp in *D. tabl*, and 19 bp in *A. djedaba* (Table 1).

In incremental order, the average base compositions of the three complete mitogenomes were 16.64% G, 25.67% T, 27.74% A, and 29.94% C (Table S1), showing that the C and A content was much higher than the G and T content. This phenomenon has also been found in other species of the family Carangidae, all of which have negative AT-skew and positive GC-skew values (Table S1), showing significant chain-specific or chain asymmetry bias. The mechanism underlying chain bias was often interpreted as evidence of asymmetrical directional mutation stress [20]. The genes with the highest average GC content were identified in *tRNA-Thr* (52.78–58.33%) and *tRNA-Ser* (50.75–57.35%) (Table 1).

### 2.2. Protein-Coding Genes (PCGs)

The 13 PCGs in the mitogenomes ranged from 165 (*ATP8*) to 1839 (*ND5*) bp (Table S2). The total length of the 13 PCGs ranged from 11,425 to 11,428 bp (Table S2), accounting for 68.99–69.05% of the entire mitogenomes. Most of the PCGs started with a typical ATG codon, except for *ATP6* (in *A. djedaba*) and *COXI* (in all three species), which started with ATA and GTG, respectively (Table 1). TAA or TAG was the termination codon in most PCGs and TAA was the most frequently used codon. However, the *CYTB*, *ND2*, *ND3*, *ND4*, *COX2*, and *COX3* genes in *A. indicus* and *D. tabl*, and the *COX2*, *ND4*, and *CYTB* genes in *A. djedaba* had incomplete termination codons TA or T (Table 1). 

**Figure 2:**
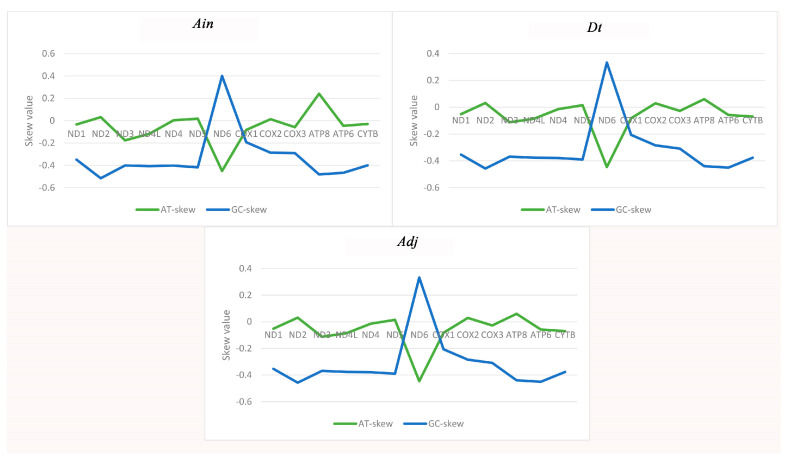
Graphical illustration showing the AT- and GC-skew in the protein-coding genes (PCGs) of the mitochondrial genome of *Alectis indicus* (*Ain*), *Decapterus tabl* (*Dt*), and *Alepes djedaba* (Adj).

The majority of the AT-skew and GC-skew values of the PCGs among the three species were negative, demonstrating that bases T and C were more plentiful than A and G (Figure 2). The lowest AT-skew and highest GC-skew values were all found in the *ND6* gene, which is unusual but similar to other observations of strand asymmetry [21,22,23]. The AT-skews for the concatenated sequence of the PCGs in all 32 species were negative, completely different from those of the entire genome. The GC-skews were all less than zero, which is identical to the complete genome (Table S1). Therefore, in view of the strange phenomenon of an AT-skew < 1 in the protein coding sequence, we analyzed the base composition of different positions of codons in the 13 PCGs, and found that the content of base T in the second codon reached 40.5% (Table S2), resulting in the increase of T content in the whole protein coding sequence, which explained the phenomenon of AT-skew < 1 in the protein coding sequence. 

**Figure 3:**
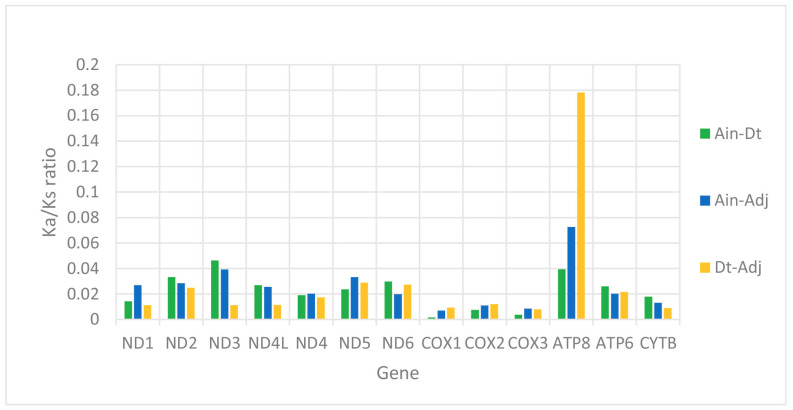
Evolutionary rates of the mitogenomes of *Alectis indicus* (Ain), *Decapterus tabl* (Dt), and *Alepes djedaba* (Adj). Note: The ratio of non-synonymous substitutions to synonymous substitutions (Ka/Ks) for each PCG is indicated.

**Figure 4:**
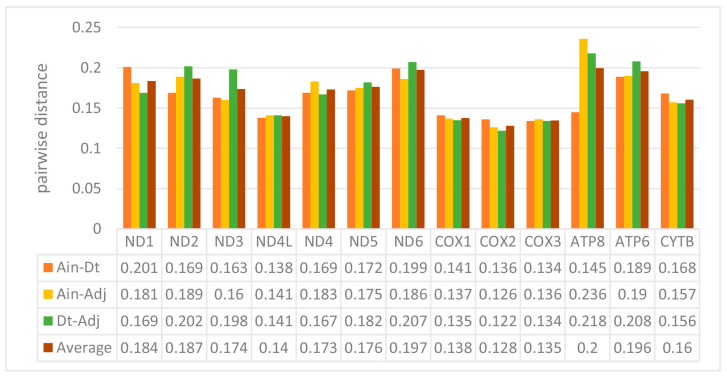
Overall mean p-genetic distances between *Alectis indicus* (*Ain*), *Decapterus tabl* (*Dt*), and *Alepes djedaba* (*Adj*) for each of the 13 PCGs. Note: Values were calculated using the first and second nucleotide positions, the entire codon, and over the full sequence.

We used the ratio of non-synonymous/synonymous mutations (Ka/Ks) value and overall p-genetic distance to describe the evolutionary rate and conservation of PCGs. The average Ka/Ks was 0.0253 in the 13 PCGs of the three Carangidae and ranged from 0.0059 (*COXI*) to 0.0967 (*ATP8*) (Figure 3). The Ka/Ks values of all PCGs were < 1, suggesting purifying selection on the functional genes [24]. In theory, purified selection would eliminate harmful mutations in the population [25]. The *ATP8* gene had the highest Ka/Ks value, and it is also found in fungi [26], flies [27], snails [28], and insects [29]. The *COXI* gene had the lowest value, which showed it was the most conserved gene. The highest overall p-genetic distances were in the *ND6* and *ATP8* genes (0.197 and 0.200, respectively), while the lowest were in the three cytochrome oxidase genes (*COXI*: 0.138, *COXII*: 0.128, *COXIII*: 0.135) (Figure 4) based on comparisons of the full-length PCGs. This suggested lowest evolutionary rates in the three cytochrome oxidase genes and higher rates in *ATP8* and *ND6*. Overall p-genetic distances once again showed that the *ATP8* gene had the highest rate of evolution and was the least conserved gene.

### 2.3. Mitochondrial Gene Codon Usage

**Figure 5:**
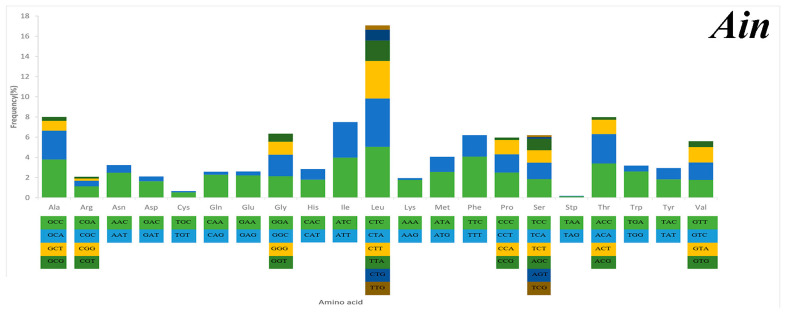

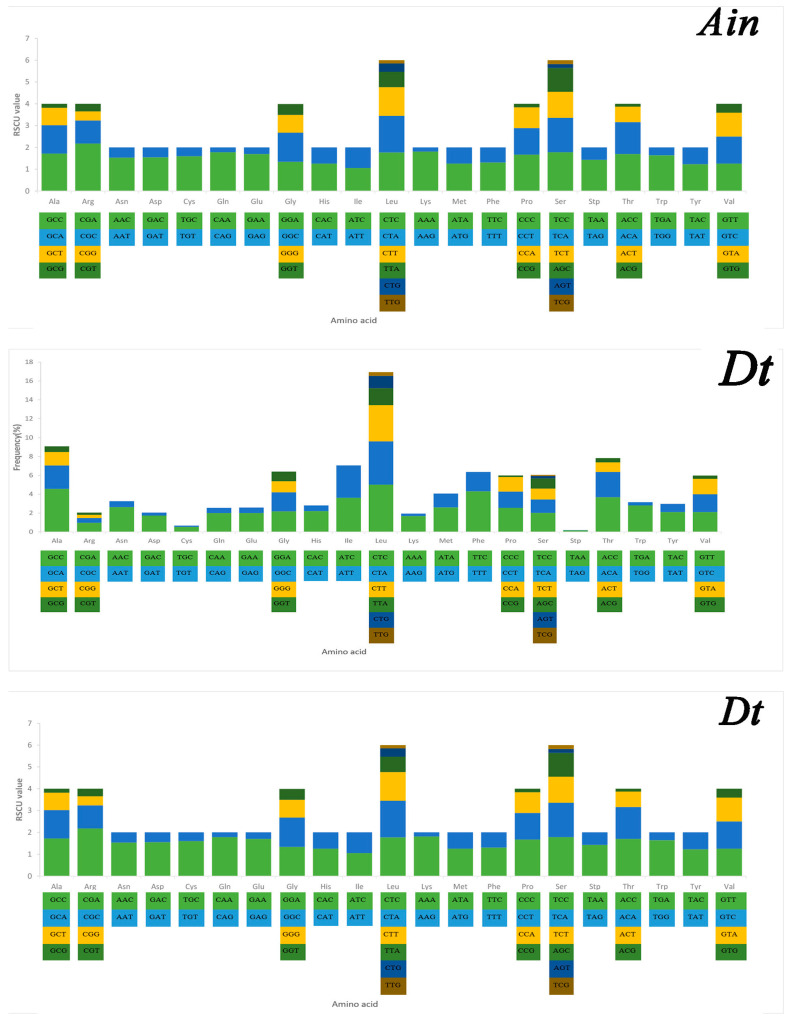

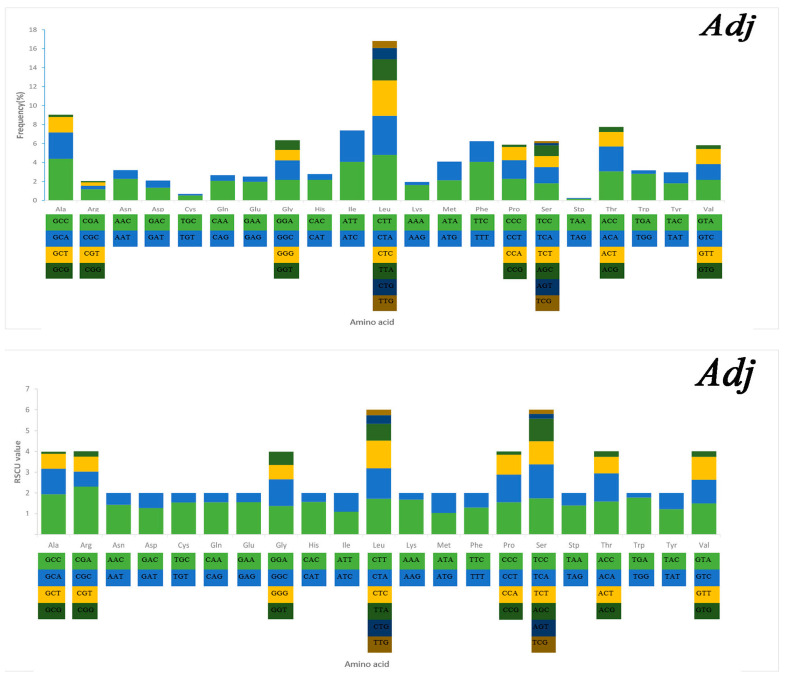
Codon frequency and RSCU (relative synonymous codon usage) in the mitochondrial genomes of *Alectis indicus* (*Ain*), *Decapterus tabl* (*Dt*), and *Alepes djedaba* (*Adj*).

Serine and leucine were encoded by six codons each, while the other amino acids were encoded by four or two codons (Figure 5). Codon frequency referred to the frequency of codon occurrence in all PCGs. Leu (CUC, CUA), Phe (UUC), Ile (AUC), and Ala (GCC) were the most frequent codons, while termination codons (UAG) and Cys (UGU) were the least frequent (Figure 5). The relative synonymous codon usage (RSCU) refers to the relative probability that a particular codon was encoded in a synonymous codon of the corresponding amino acid. Arg (CGA), Lys (AAA), Gln (CAA), and Ser (TCC) were the most frequent, while Ser (AGT, TCG), Thr (ACG), Leu (TTG), Ala (GCG), and Pro (CCG) were rare in the RSCU analysis (Figure 5). The chain asymmetry caused by the difference in base composition could also affect codon usage. C and A bases were more frequent in the third positions of codons than were U and G bases (Table S2), which showed that the codons NNA and NNC were a minority, whereas the synonymous codons NNU and NNG were the majority.

### 2.4. Ribosomal and Transfer RNA Genes

The length of the *12S rRNA* gene ranged from 951 to 954 bp, while the *16S rRNA* gene ranged from 1716 to 1728 bp (Table 1). Consistent with most Osteichthyes, the *12S* and *16S rRNA* genes were separated by *tRNA-Val* and located between the *tRNA-Leu* and *tRNA-Phe* genes. The AT content ranged from 52.53% to 53.77% (Table S1). The average AT- and GC-skews of these two rRNA genes were 0.178 to 0.205 and −0.119 to −0.082, respectively (Table S2).

Twenty two tRNA genes were identified in the mitogenomes of the three species (Table 1). Their sizes ranged from 66 (*tRNA-Cys*) to 75 (*tRNA-Leu* and *tRNA-Lys*) bp. The distribution of nucleotides was not identical to the rest of the entire mitogenome: the rRNAs and PCGs had a negative GC-skew value, while the 22 tRNA genes had a positive GC-skew value from 0.035 to 0.055 among the three species (Table S2). Codons and anticodons typically showed one-to-one correspondence. However, leucine was determined by two anticodons (UAG and UAA) and serine was determined by UGA and GCU in the three species (Table 1). The typical cloverleaf secondary structure was predicted for all tRNA genes of the three species, except *tRNA-Ser* (GCT), although many U-G base pairs and non-complementary pairs existed in the stem regions. According to a previous report, stem mismatches were repaired by post-transcriptional editing and were common in mitochondrial tRNA genes [30]. There was no recognizable DHU stem in *tRNA-Ser* (GCT) in the mitogenomes of the three species, which is common in most vertebrate mitogenomes [31,32]. Nevertheless, it functioned like typical tRNAs to fit the ribosomes by adjusting its structural conformation [33].

### 2.5. CGView Comparison Tool (CCT) Map

**Figure 6:**
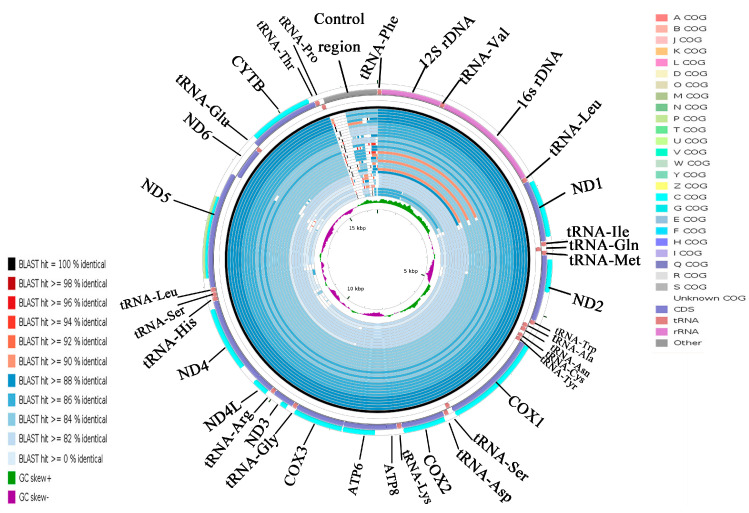
Graphical map of the blast results showing the nucleotide identity between the complete mitogenomes of *Alectis indicus* and 31 other Carangidae using the CGView comparison tool (CCT). CCT arranges blast results in order so that sequences that are most similar to the reference (*A. indicus*) are placed closer to the outer edge of the map. Note: Clusters of orthologous groups of proteins (COG), gene region, blast identity, and AT-skew are shown from outside to inside. The species from outside to inside are as follows: *Alectis indicus*, *Alectis ciliaris*, *Uraspis secunda*, *Uraspis helvola*, *Parastromateus niger*, *Carangoides armatus*, *Carangoides malabaricus*, *Trachurus trachurus*, *Trachurus japonicus*, *Megalaspis cordyla*, *Caranx melampygus*, *Decapterus macrosoma*, *Decapterus tabl*, *Decapterus macarellus*, *Caranx tille*, *Alepes kleinii*, *Decapterus maruadsi*, *Alepes djedaba*, *Atule mate*, *Caranx ignobilis*, *Selaroides leptolepis*, *Selar crumenophthalmus*, *Kaiwarinus equula*, *Elagatis bipinnulata*, *Seriola rivoliana*, *Seriola quinqueradiata*, *Seriola dumerili*, *Seriolina nigrofasciata*, *Seriola lalandi*, *Trachinotus blochii*, *Trachinotus carolinus*, and *Trachinotus ovatus*.

Using the *A. indicus* mitogenome as the reference sequence, all available mitogenomes in the family Carangidae were compared using CCT. This visual map showed that the differences of the species sequence of the family Carangidae mainly came from the control region sequence, and the other parts of the sequences were very similar (Figure 6). *A. indicus* was most closely related to *A. ciliaris* and then with *Uraspis secunda* and *U. helvola* (Figure 6). The identity in the whole genome sequence was lower than that of the PCGs (Figure 6 and Figure S2). *COXI* was the most conserved gene, which makes it suitable for evolutionary analyses and molecular phylogenetics in the family Carangidae. By contrast, *ATP8* was the least conserved gene in all species. The result was consistent with the previous analysis of Ka/Ks and overall p-genetic distance.

Cluster of orthologous groups (COG) could indicate the presence of certain proteins in a given species in a particular PCG, and could be used to determine whether a particular metabolic pathway exists in a species [34]. Twelve COGs were detected in 11 PCGs, excluding *ATP8* and *ND6* (Figure S1). *ND5* had two COGs (p COG and c COG), and each of the other 10 PCGs had a single COG (c COG). Each type of COG had its own unique functions [35]. The functions of the 12 COGs identified were related to metabolism. The p COG participated in inorganic ion transport and metabolism while the c COG participated in energy production and transformation [36]. The COGs in *ND5* indicated that *ND5* is involved in two processes: inorganic ion transport and metabolism and energy production and conversion.

### 2.6. Phylogenetic Analyses

**Figure 7:**
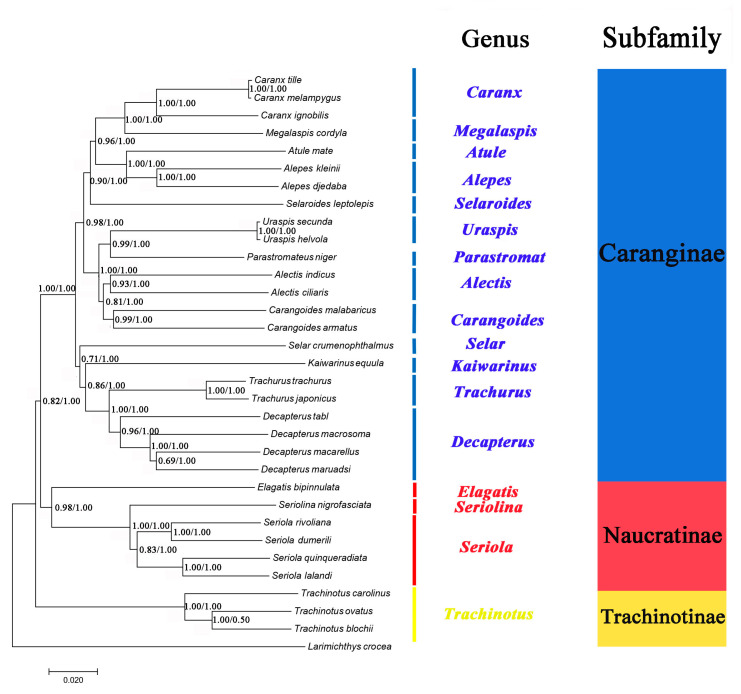
Phylogenetic tree inferred from the amino acid sequences of 13 PCGs of 32 species of Carangidae. *Larimichthys crocea* was used as the outgroup. The numbers at the nodes are the bootstrap support values (**left**) and Bayesian posterior probabilities (**right**).

The phylogenetic relationships of 32 Carangidae species were reconstructed based on the concatenated sequence of their mitochondrial PCGs genes (Figure 7). Phylogenetic trees inferred from the maximum likelihood (ML) and Bayesian inference (BI) analyses had largely identical topological structure and are illustrated together in Figure 7. The topology produced three clades corresponding to the subfamilies Caranginae, Naucratinae, and Trachinotinae. The monophyly of each subfamily was well supported with high branch support values (ML bootstrap values > 0.85, BI posterior probabilities = 1.00). The phylogenetic relationships support the groupings (Trachinotinae + (Naucratinae + Caranginae)), which were consistent with morphological taxonomy [37] and previous molecular systematics studies based on single mitochondrial gene sequences [18,19]. The intergeneric and interspecific taxonomic positions were also clearly resolved within each of the subfamilies. *A. indicus* was most closely related to *A. ciliaris*, and they formed a group (genus *Alectis*) that was a sister to genus *Carangoides*. *D. tabl* was on the same branch with the other *Decapterus* species and formed a sister group with genus *Trachurus*. *A. djedaba* together with *A. kleinii* formed a group (genus *Alepes*) with the genus *Atule* as sister to them.

Furthermore, *Kaiwarinus equula* is considered a synonym of *Carangoides equula* according to the current taxonomic system [38]. However, phylogenetic analysis in this study showed that *K. equula* and *Carangoides* spp. were on the different branches (Figure 7), supporting that *K. equula* belongs to an independent genus and it should be the accepted name instead of *C. equula*.

### 2.7. Estimation of Divergence Times

**Figure 8:**
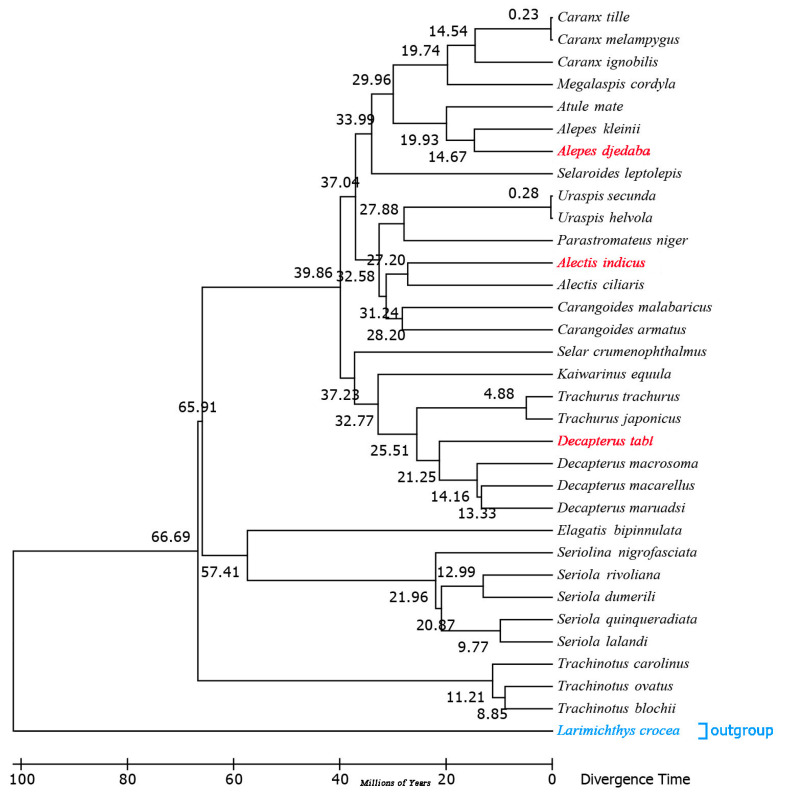
Chronogram for the 32 species of Carangidae with a single outgroup *Larimichthys crocea* based on the Bayesian topology calculated from analysis of the 13 PCGs. Divergence times were estimated using three calibrations. The red fonts represent the three species sequenced in this study, and the blue fonts represent the outgroup species.

The divergence timescale within Carangidae was estimated based on phylogenetic trees by using the RelTime-ML method. The divergence times showed that *A. indicus* began to differentiate from other species about 27.20 million years ago (Mya) within the early Miocene, while *D. tabl,* 21.25 Mya, and *A. djedaba,* 14.67 Mya, did so in the middle Oligocene (Figure 8 and Figure S2). The Carangidae began to differentiate at the end of the Cretaceous, about 79 Mya, when the Cretaceous–Paleogene extinction event happened. The split between Trachinotinae and (Naucratinae + Caranginae) was 66.69 Mya within the middle Paleocene epoch. The divergence time of the subfamily Naucratinae and Caranginae could be dated back to 65.91 Mya. Most of the species differentiated in the Paleogene (Figure 8 and Figure S2), supporting previous findings that the Paleogene was the heyday of species evolution [39]. This phenomenon might be due to the huge environmental changes caused by the Cretaceous mass extinction, which led to the outbreak of species diversity [40]. Previous studies based on Cytb sequences showed that the genera *Trachurus* and *Seriola* [41] began to differentiate at 20.1 and 55 Mya, respectively. In this study, the full mitogenome sequence analysis indicated that the genera *Trachurus* and *Seriola* began to differentiate at 25.51 and 57.41 Mya (Figure S2), which is a little earlier than the previous estimates.

## 3. Materials and Methods

### 3.1. Sample Collection and DNA Extraction

Wild samples of *A. indicus*, *D. tabl*, and *A. djedaba* were captured in the South China Sea at 22°48′ N, 113°07′ E (7 July 2014); 23°11′ N, 110°30′ E (24 April 2014); and 23°58′ N, 110°02′ E (1 March 2015), respectively. The dorsal muscle was harvested and returned to the laboratory in anhydrous alcohol, and genomic DNA was extracted using a DNeasy Blood and Tissue kit (QIAGEN, (Qiagen, Venlo, The Netherlands) following the manufacturer’s protocol. DNA was quantified by ultra-micro spectrophotometry and gel electrophoresis. All animal experiments were conducted in accordance with the guidelines and approval of the Animal Research and Ethics Committees of South China Agricultural University. 

### 3.2. Sequencing, Assembly, and Annotation

A library was constructed on the Illumina platform. High-quality DNA samples were randomly broken into fragments for paired-end sequencing, and the DNA libraries were constructed according to the procedure of Illumina DNA library construction. After selecting the fragments, finishing the ends, and adding poly A, adapters were added to both ends of the DNA fragment with ligase. The ligated product was amplified, selected, and purified. Finally, a 100 bp library was generated for sequencing on the Illumina HiSeq2500 instrument [42].

The SOAPdenovo2 (BGI, Shenzhen, China) software package was used for de novo assembly of the mitogenomes [43]. OGDRAW 1.3.1 (Max Planck Institute of Molecular Plant Physiology, Potsdam-Golm, Germany) was used to draw a mitochondrial genetic map [44]. The PCGs and ribosomal genes were identified by blast [45]. The rules for the vertebrate mitochondria genetic code were used [46]. The structure of the transfer RNA genes was predicted with tRNA scan-SE ver. 1.3.1 (Washington University School of Medicine, St Louis, Missouri, USA) and confirmed with MitoFish (http://mitofish.aori.u-tokyo.ac.jp/) [47].

### 3.3. Sequence Analyses

The mitochondrial strand asymmetry was assessed using the formulas: GC-skew = [G − C] / [G + C] and AT-skew = [A − T] / [A + T] [48]. The tandem repeat finder Dot2dot was used to identify repeat sequences [49]. Codon frequency referred to the occurrence frequency of codons in all PCGs and relative synonymous codon usage (RSCU) referred to the relative probability that a particular codon was encoded in the synonymous codon of the corresponding amino acid. The codon frequency and RSCU values were calculated with MEGA 7.0 (Tokyo Metropolitan University, Tokyo, Japan) [50] and both indicate codon usage [51]. The RSCU values were independent of the amino acid usage, and the codon abundance directly reflected the degree of preference of codon usage [52]. If there was no preference for codon use, the RSCU value of the codon was equal to 1. When the RSCU value of a codon was > l, it indicated that the codon was used relatively frequently and vice versa.

### 3.4. Phylogenetic Analyses

The concatenated sequences of 13 PCGs (without stop codons) extracted from the reported 32 mitogenomes of Carangidae species, including *A. indicus*, *D. tabl*, and *A. djedaba,* were used to construct phylogenetic trees with MEGA 7.0, MrBayes 3.1 (University of California, La Jolla, San Diego, CA, USA) [53], and iTOL (https://itol.embl.de/) [54], using *Larimichthys crocea* (GenBank accession number: EU339149) as the outgroup. Bayesian Inference (BI) was analyzed in MrBayes 3.1 [55] and maximum likelihood (ML) analysis was performed in MEGA 7.0. When performing Bayesian analysis, general time reversible (GTR) was adopted in the data substitution model and the ratio of the difference between sites was set as invgamma. The Markov chain Monte Carlo method was used to estimate the posterior probability by calculating 10,000 generations, sampling once every 10 generations, and discarding 250 aged samples from the obtained samples to summarize and establish a shared tree. The standard deviation of the split frequency of the BI tree was less than 0.01 to guarantee the BI tree was reliable. The ML tree was constructed with 1000 bootstrap replicates. DeepFin (http://deepfin.org/) [56] and the NCBI taxonomy server [57] were used for further analyses.

### 3.5. Divergence Times

Divergence times of the 32 Carangidae were estimated with the GTR + G + I modeling and RelTime-ML method with MEGA 7.0 [58]. The TimeTree was computed using two calibration constraints. In addition, the divergence times of 27 genera of Carangidae were estimated using the TimeTree database [59]. To ensure consistency between the divergence times and phylogenetic tree, the divergence times were generated by importing the Bayesian phylogenetic tree into MEGA 7.0. The CGView comparison tool was used to draw a circular genetic similarity map of Carangidae using *A. indicus* mitogenome sequence as the reference sequence [60].

## 4. Conclusions

The study characterized the complete mitogenomes of three Carangidae species (i.e., *Alectis indicus*, *Decapterus tabl*, and *Alepes djedaba*). They are composed of 37 genes comprising two rRNA genes, 22 tRNA genes, 13 protein-coding genes, and a non-coding region. Comprehensive analyses of Ka/Ks, overall p-genetic distance, and a CCT map demonstrated the highest evolutionary rate in ATP8, while COXI/COXII appear to have the lowest evolutionary rate. The phylogenetic trees inferred from the maximum likelihood and Bayesian inference using mitochondrial PCGs genes supported three clades correspondent to subfamily and the groupings (Trachinotinae + (Naucratinae + Caranginae)) and also provided support for the monophyly of these subfamilies. However, complete mitogenome sequences from Scomberoidinae, the fourth subfamily in Carangidae, were absent in the phylogenetic analyses, making their position still unclear. More sampling of Scomberoidinae will be needed for a thorough phylogenetic analysis of the major groups within Carangidae.

## Figures and Tables

**Table 1:** Summary of gene/element features of *Alectis indicus, Decapterus tabl*, and *Alepes djedaba*.

Gene Element	Strand	Size (bp)	GC Content (%)	Amino Acids (aa)	Inferred Initiation Codon	Inferred Termination Codon	One Letter Code	Anti-Codon	Intergenic Nucleotide*(bp)
*tRNA-Phe*	H	68	45.59–51.47				F	GAA	0
*12S rDNA*	H	951–954	47.79–49.21						0
*tRNA-Val*	H	72	47.22–50.00				V	TAC	0
*16S rDNA*	H	1716–1728	45.37–46.50						0
*tRNA-Leu*	H	74–75	48.00–48.65				L	TAA	0
*ND1*	H	975	48.82–51.08	324	ATG	TAG, TAA			0
*tRNA-Ile*	H	70	50.00–52.86				I	GAT	5–6
*tRNA-Gln*	L	71	40.85				Q	TTG	−1
*tRNA-Met*	H	70–71	45.71–47.14				M	CAT	−1
*ND2*	H	1045–1047	49.00–50.43	348	ATG	T, TAG			0
*tRNA-Trp*	H	69–71	50.70–52.17				W	TCA	0
*tRNA-Ala*	L	69	39.13–43.48				A	TGC	1
*tRNA-Asn*	L	73	47.95–52.05				N	GTT	1
*tRNA-Cys*	L	66–67	43.28–45.45				C	GCA	37–38
*tRNA-Tyr*	L	70	45.71–47.14				Y	GTA	0
*COX1*	H	1551	45.07–47.58	516	GTG	TAA			1
*tRNA-Ser*	L	71	46.48–47.89				S	TGA	0
*tRNA-Asp*	H	71	43.66–46.48				D	GTC	3
*COX2*	H	691	41.68–44.86	230	ATG	T			6–7
*tRNA-Lys*	H	74–75	45.67–48.00				K	TTT	0
*ATP8*	H	165–168	44.64–49.70	55	ATG	TAA, TAG			1
*ATP6*	H	678–684	44.84–50.00	225–227	ATG or ATA	TAA			(−10)–(−4)
*COX3*	H	785–786	47.46–49.17	260–261	ATG	TA, TAA			−1
*tRNA-Gly*	H	69–70	32.86–36.23				G	TCC	0
*ND3*	H	349–351	47.85–49.00	115–116	ATG	T, TAG			0
*tRNA-Arg*	H	67-69	29.85-37.68				R	TCG	0
*ND4L*	H	297	48.82–52.86	98	ATG	TAA			1
*ND4*	H	1381	46.85–49.17	460	ATG	T			−7
*tRNA-His*	H	71–73	36.99–39.44				H	GTG	0
*tRNA-Ser*	H	67–68	50.75–57.35				S	GCT	0
*tRNA-Leu*	H	73	45.21				L	TAG	4
*ND5*	H	1839	44.54–47.80	612	ATG	TAA, TAG			0
*ND6*	L	522	44.44–48.85	173	ATG	TAG, TAA			−4
*tRNA-Glu*	L	69	40.58–43.48				E	TTC	0
*CYTB*	H	1141	46.71–49.69	380	ATG	T			4
*tRNA-Thr*	H	72	52.78–58.33				T	TGT	0
*tRNA-Pro*	L	71	38.03–45.07				P	TGG	−1

## Supplementary Materials

Supplementary materials can be found at https://www.mdpi.com/1422-0067/21/13/4685/s1.

## References

[1] Boore J.L. (1999). Animal mitochondrial genomes. Nucleic Acids Res..

[2] Wolstenholme D.R. (1992). Animal Mitochondrial DNA: Structure and Evolution. Int. Rev. Cytol..

[3] Ceruso M., Mascolo C., Anastasio A., Pepe T., Sordino P. (2019). Frauds and fish species authentication: Study of the complete mitochondrial genome of some Sparidae to provide specific barcode markers. Food Control.

[4] Sousa-Santos C., Pereira A., Branco P., Costa G., Santos J., Ferreira M., Lima C., Doadrio I., Robalo J. (2018). Mito-nuclear sequencing is paramount to correctly identify sympatric hybridizing fishes. Acta Ichthyol. Piscat..

[5] Chen K.-C., Zakaria D., Altarawneh H., Andrews G.N., Ganesan G.S., John K.M., Khan S., Ladumor H. (2019). DNA barcoding of fish species reveals low rate of package mislabeling in Qatar. Genome.

[6] Schroeter J.C., Maloy A.P., Rees C.B., Bartron M.L. (2019). Fish mitochondrial genome sequencing: Expanding genetic resources to support species detection and biodiversity monitoring using environmental DNA. Conserv. Genet. Resour..

[7] Rincón-Sandoval M., Betancur-R R., Maldonado-Ocampo J.A. (2019). Comparative phylogeography of trans-Andean freshwater fishes based on genome-wide nuclear and mitochondrial markers. Mol. Ecol..

[8] Saad Y. (2019). Analysis of 16S mitochondrial ribosomal DNA sequence variations and phylogenetic relations among some Serranidae fishes. South Afr. J. Anim. Sci..

[9] Mirande J.M. (2018). Morphology, molecules and the phylogeny of Characidae (Teleostei, Characiformes). Cladistics.

[10] Betancur-R R., Broughton R.E., Wiley E.O., Carpenter K., López J.A., Li C., Holcroft N.I., Arcila D., Sanciangco M., Ii J.C.C. (2013). The Tree of Life and a New Classification of Bony Fishes. PLoS Curr..

[11] Betancur-R R., Wiley E.O., Arratia G., Acero A., Bailly N., Miya M., Lecointre G., Orti G. (2017). Phylogenetic classification of bony fishes. BMC Evol. Boil..

[12] Cárdenas L., Hernández C.E., Poulin E., Magoulas A., Kornfield I., Ojeda F.P. (2005). Origin, diversification, and historical biogeography of the genus Trachurus (Perciformes: Carangidae). Mol. Phylogenet. Evol..

[13] Olusola O. (2011). Technological properties and proximate composition of two imported fish species in Nigeria. EJEAFChe Electron. J. Environ. Agric. Food Chem..

[14] Hosseinzadeh Sahafi H. (2000). Identification of marine ornamental fishes in northern part of the Persian Gulf. Iran. J. Fish. Sci..

[15] Edwards R.R.C., Bakhader A., Shaher S. (1985). Growth, mortality, age composition and fisheries yields of fish from the Gulf of Aden. J. Fish Boil..

[16] Ohshimo S., Shiraishi T., Tanaka H., Yasuda T., Yoda M., Ishida H., Tomiyasu S. (2014). Growth and Reproductive Characteristics of the Roughear Scad Decapterus tabl in the East China Sea. Jpn. Agric. Res. Q. JARQ.

[17] Siwat V., Ambariyanto A., Widowati I. (2016). Biometrics of bigeye scad, Selar crumenophthalmus and shrimp scad, Alepes djedaba from Semarang waters, Indonesia. Aquacult. Aquarium Conserv. Legis..

[18] Reed D.L., E Carpenter K., Degravelle M.J. (2002). Molecular systematics of the Jacks (Perciformes: Carangidae) based on mitochondrial cytochrome b sequences using parsimony, likelihood, and Bayesian approaches. Mol. Phylogenet. Evol..

[19] Damerau M., Freese M., Hanel R. (2017). Multi-gene phylogeny of jacks and pompanos (Carangidae), including placement of monotypic vadigo Campogramma glaycos. J. Fish Boil..

[20] Tomkova M., Tomek J., Kriaucionis S., Schuster-Böckler B. (2018). Mutational signature distribution varies with DNA replication timing and strand asymmetry. Genome Boil..

[21] Lü Z., Zhu K., Jiang H., Lu X., Liu B., Ye Y., Jiang L., Liu L., Gong L. (2019). Complete mitochondrial genome of Ophichthus brevicaudatus reveals novel gene order and phylogenetic relationships of Anguilliformes. Int. J. Boil. Macromol..

[22] Mu X., Liu Y., Lai M., Song H., Wang X., Hu Y., Luo J. (2015). Characterization of the Macropodus opercularis complete mitochondrial genome and family Channidae taxonomy using Illumina-based de novo transcriptome sequencing. Gene.

[23] Yang H., Xia J., Zhang J.-E., Yang J., Zhao H., Wang Q., Sun J., Xue H., Wu Y., Chen J. (2018). Characterization of the Complete Mitochondrial Genome Sequences of Three Croakers (Perciformes, Sciaenidae) and Novel Insights into the Phylogenetics. Int. J. Mol. Sci..

[24] Nielsen R., Yang Z. (1998). Likelihood models for detecting positively selected amino acid sites and applications to the HIV-1 envelope gene. Genetics.

[25] García G.O., Oteo J.A. (2019). Evolutionary distances corrected for purifying selection and ancestral polymorphisms. J. Theor. Boil..

[26] Li Q., Wang Q., Chen C., Jin X., Chen Z., Xiong C., Li P., Zhao J., Huang W. (2018). Characterization and comparative mitogenomic analysis of six newly sequenced mitochondrial genomes from ectomycorrhizal fungi (Russula) and phylogenetic analysis of the Agaricomycetes. Int. J. Boil. Macromol..

[27] Huang J., Ma T. (2018). Comparative analysis of two mitochondrial genomes of flesh flies (Sarcophaga antilope and Sarcophaga dux) with phylogeny and evolutionary timescale for Sarcophagidae. Int. J. Boil. Macromol..

[28] Yang H., Zhang J.-E., Xia J., Yang J., Guo J., Deng Z., Luo M. (2018). Comparative Characterization of the Complete Mitochondrial Genomes of the Three Apple Snails (Gastropoda: Ampullariidae) and the Phylogenetic Analyses. Int. J. Mol. Sci..

[29] Zhang D.-L., Gao J., Li M., Yuan J., Liang J., Yang H., Bu W. (2019). The complete mitochondrial genome of Tetraphleps aterrimus (Hemiptera: Anthocoridae): Genomic comparisons and phylogenetic analysis of Cimicomorpha. Int. J. Boil. Macromol..

[30] Lavrov D., Brown W.M., Boore J.L. (2000). A novel type of RNA editing occurs in the mitochondrial tRNAs of the centipede Lithobius forficatus. Proc. Natl. Acad. Sci. USA.

[31] Cui P., Jirimutu Z., Ding F., Qi D., Gao H., Menghe B., Yu J., Hu S., Zhang H. (2007). A complete mitochondrial genome sequence of the wild two-humped camel (Camelus bactrianus ferus): An evolutionary history of camelidae. BMC Genom..

[32] Zhou Y., Zhang J.-Y., Zheng R., Yu B.-G., Yang G. (2009). Complete nucleotide sequence and gene organization of the mitochondrial genome of Paa spinosa (Anura: Ranoidae). Gene.

[33] Ohtsuki T., Kawai G., Watanabe K. (2002). The minimal tRNA: Unique structure of Ascaris suum mitochondrial tRNASer UCU having a short T arm and lacking the entire D arm. FEBS Lett..

[34] Remm M., Storm C.E., Sonnhammer E. (2001). Automatic clustering of orthologs and in-paralogs from pairwise species comparisons. J. Mol. Boil..

[35] A Natale D., Shankavaram U.T., Galperin M.Y., I Wolf Y., Aravind L., Koonin E.V. (2000). Towards understanding the first genome sequence of a crenarchaeon by genome annotation using clusters of orthologous groups of proteins (COGs). Genome Boil..

[36] Meyers R. (2019). Children’s Oncology Group (COG) Surgery Discipline Committee: Research Agenda and Collaborations. Pediatr. Blood Cancer.

[37] Gushiken S. (1988). Phylogenetic Relationships of the Perciform Genera of the Family Carangidae. Ichthyol. Res..

[38] FishBase. https://www.fishbase.org.version.

[39] Miya M., Friedman M., Satoh T.P., Takeshima H., Sado T., Iwasaki W., Yamanoue Y., Nakatani M., Mabuchi K., Inoue J. (2013). Evolutionary Origin of the Scombridae (Tunas and Mackerels): Members of a Paleogene Adaptive Radiation with 14 Other Pelagic Fish Families. PLoS ONE.

[40] Zinsmeister W.J. (1998). Discovery of fish mortality horizon at the K-T Boundary on Seymour Island: Re-evaluation of events at the end of the Cretaceous. J. Paléontol..

[41] Swart B.L., Von Der Heyden S., Der Merwe A.B.-V., Roodt-Wilding R. (2015). Molecular systematics and biogeography of the circumglobally distributed genus Seriola (Pisces: Carangidae). Mol. Phylogenet. Evol..

[42] Mak S.S.T., Gopalakrishnan S., Carøe C., Geng C., Liu S., Sinding M.-H.S., Kuderna L.F.K., Zhang W., Fu S., Vieira F.G. (2017). Comparative performance of the BGISEQ-500 vs Illumina HiSeq2500 sequencing platforms for palaeogenomic sequencing. GigaScience.

[43] Bankevich A., Nurk S., Antipov D., Gurevich A.A., Dvorkin M., Kulikov A.S., Lesin V.M., Nikolenko S.I., Pham S., Prjibelski A.D. (2012). SPAdes: A New Genome Assembly Algorithm and Its Applications to Single-Cell Sequencing. J. Comput. Biol..

[44] Greiner S., Lehwark P., Bock R. (2019). OrganellarGenomeDRAW (OGDRAW) version 1.3.1: Expanded toolkit for the graphical visualization of organellar genomes. Nucleic Acids Res..

[45] Chang C.-Y., Li Y.-C., Chen N.-C., Huang X.-X., Lu Y.-C. In A special processor design for nucleotide basic local alignment search tool with a new banded two-hit method. Proceedings of the 2016 IEEE Nordic Circuits and Systems Conference (NORCAS).

[46] Zhang Q.-L., Yang X.-Z., Zhang L., Feng R.-Q., Zhu Q.-H., Chen J.-Y., Yuan M.-L. (2018). Adaptive evidence of mitochondrial genomes in Dolycoris baccarum (Hemiptera: Pentatomidae) to divergent altitude environments. Mitochondrial DNA Part A.

[47] Iwasaki W., Fukunaga T., Isagozawa R., Yamada K., Maeda Y., Satoh T.P., Sado T., Mabuchi K., Takeshima H., Miya M. (2013). MitoFish and MitoAnnotator: A mitochondrial genome database of fish with an accurate and automatic annotation pipeline. Mol. Boil. Evol..

[48] Junqueira A.C.M., Lessinger A.C., Torres T.T., Da Silva F.R., Vettore A.L., Arruda P., Espin A.M.L.A. (2004). The mitochondrial genome of the blowfly Chrysomya chloropyga (Diptera: Calliphoridae). Gene.

[49] Genovese L.M., Mosca M.M., Pellegrini M., Geraci F. (2019). Dot2dot: Accurate whole-genome tandem repeats discovery. Bioinformatics.

[50] Kumar V., Dey A., Singh A. (2009). MEGA: A Bio Computational Software for Sequence and Phylogenetic Analysis. Lect. Notes Eng. Comput. Sci..

[51] Kumar S., Stecher G., Tamura K. (2016). MEGA7: Molecular Evolutionary Genetics Analysis Version 7.0 for Bigger Datasets. Mol. Biol. Evol..

[52] Vadimirov N.V., Kochetov A.V., Grigorovich D.A., Matushkin Y.G. (2006). RSCU_comparer: A new statistical tool for practical analysis of codon usage. Proceedings of the Fifth International Conference on Bioinformatics of Genome Regulation And Structure. Comp. Evol. Genom. Proteom..

[53] Ronquist F., Huelsenbeck J.P. (2003). MrBayes 3: Bayesian phylogenetic inference under mixed models. Bioinformatics.

[54] Letunic I., Bork P. (2019). Interactive Tree Of Life (iTOL) v4: Recent updates and new developments. Nucleic Acids Res..

[55] Pang S., Stones R.J., Ren M., Liu X.-G., Wang G., Xia H.-J., Wu H.-Y., Liu Y., Xie Q. (2015). GPU MrBayes V3.1: MrBayes on Graphics Processing Units for Protein Sequence Data: Table 1. Mol. Boil. Evol..

[56] Tan M., Armbruster J.W. (2018). Phylogenetic classification of extant genera of fishes of the order Cypriniformes (Teleostei: Ostariophysi). Zootaxa.

[57] Sadjjadi S.M., Ebrahimipour M., Sadjjadi S.M. (2019). Comparison between Echinococcus granulosus sensu stricto (G1) and E. canadensis (G6) mitochondrial genes (cox1 and nad1) and their related protein models using experimental and bioinformatics analysis. Comput. Boil. Chem..

[58] Mello B. (2018). Estimating TimeTrees with MEGA and the TimeTree Resource. Mol. Boil. Evol..

[59] Kumar S., Stecher G., Suleski M., Hedges S.B. (2017). TimeTree: A Resource for Timelines, Timetrees, and Divergence Times. Mol. Boil. Evol..

[60] Grant J.R., Arantes A.S., Stothard P. (2012). Comparing thousands of circular genomes using the CGView Comparison Tool. BMC Genom..

