# Supplementary material for: Complete Mitogenomes of Three Carangidae (Perciformes) Fishes: Genome Description and Phylogenetic Considerations
Source: Int J Mol Sci. 2020 Jun 30;21(13):4685. doi: 10.3390/ijms21134685 (PMC7370159; doi:10.3390/ijms21134685)
Supplement: Supplementary file 1 [file ijms-21-04685-s001.pdf]

International Journal of *Molecular Sciences*

MDPI

Supplementary Materials:

Table S1. Summary of the base composition of the mitogenomes at each codon position of the concatenated 13 protein-coding genes (PCGs) across 32 species in the family Carangidae.

Species	Accession Number	Entire Genome							Protein-Coding Genes		
		Length (bp)	A(%)	G(%)	T(%)	C(%)	AT-Skew	GC-Skew	Length (bp)	AT-Skew	GC-Skew
Alectis Indicus	KP710215	16553	28.03	16.21	25.60	30.16	0.0453	-0.3008	3800	-0.0424	-0.3377
Decapterus tabl	MN102718	16545	27.28	17.05	25.02	30.65	0.0432	-0.2851	3799	-0.0480	-0.3250
Alepes djedaba	KP408222	16563	27.92	16.66	26.40	29.03	0.0279	-0.2707	3798	-0.0654	-0.3080
Alectis ciliaris	NC_025566	16570	28.32	16.16	26.77	28.75	0.0281	-0.2803	3640	-0.0597	-0.3183
Alepes kleinii	NC_023524	16571	28.06	16.45	27.02	28.47	0.0188	-0.2675	3651	-0.0685	-0.3049
Atule mate	NC_026222	16565	28.37	16.29	27.62	27.72	0.0133	-0.2597	3690	-0.0819	-0.2909
Carangoides armatus	NC_004405	16556	28.03	16.09	26.50	29.37	0.0280	-0.2921	3573	-0.0574	-0.3342
Carangoides malabaricus	NC_023968	16561	27.83	16.38	26.15	29.64	0.0311	-0.2881	3667	-0.0558	-0.3191
Caranx ignobilis	NC_022932	16588	28.83	16.05	25.81	29.30	0.0552	-0.2921	3637	-0.0334	-0.3232
Caranx melampygus	NC_004406	16593	28.95	15.84	26.28	28.93	0.0483	-0.2923	3568	-0.0324	-0.3299
Caranx tille	NC_029421	16593	28.95	15.84	26.26	28.95	0.0487	-0.2926	3633	-0.0316	-0.3303
Decapterus macrosoma	NC_023458	16545	27.05	17.17	25.39	30.39	0.0316	-0.2779	3674	-0.0569	-0.3173
Decapterus macarellus	NC_026718	16544	27.27	17.03	25.27	30.43	0.0380	-0.2822	3657	-0.0472	-0.3232
Decapterus maruadsi	NC_024556	16541	28.95	15.84	26.28	28.93	0.0483	-0.2923	3651	-0.0479	-0.3259
Elagatis bipinnulata	NC_029880	16542	27.93	16.76	25.81	29.49	0.0394	-0.2751	3639	-0.0475	-0.3093
Kaiwarinus equula	NC_025644	16588	26.31	18.13	25.33	30.24	0.0189	-0.2503	3652	-0.0797	-0.2745
Megalaspis cordyla	NC_025565	16566	28.83	15.93	25.81	29.43	0.0552	-0.2976	3637	-0.0275	-0.3299
Parastromateus niger	KJ192332	16561	28.32	16.16	26.01	29.51	0.0425	-0.2923	3656	-0.0445	-0.3268
Selar crumenophthalmus	NC_023954	16610	27.18	16.77	26.57	29.48	0.0113	-0.2748	3648	-0.0706	-0.3117
Selaroides leptolepis	NC_029184	16560	27.77	16.76	26.53	28.94	0.0228	-0.2665	3624	-0.0613	-0.2964
Seriola dumerili	NC_016870	16530	26.84	17.60	25.50	30.05	0.0256	-0.2612	3661	-0.0586	-0.2961
Seriola lalandi	NC_016869	16535	26.67	17.80	25.34	30.20	0.0255	-0.2583	3643	-0.0614	-0.2912
Seriola quinqueradiata	NC_016868	16539	26.59	18.03	25.19	30.20	0.0270	-0.2523	3660	-0.0644	-0.2788
Seriola rivoliana	NC_027183	16599	27.30	17.15	25.74	29.80	0.0294	-0.2694	3656	-0.0502	-0.3064
Seriolina nigrofasciata	NC_028420	16531	26.67	17.50	25.84	29.99	0.0158	-0.2630	3656	-0.0627	-0.3000
Trachinotus blochii	NC_024026	16558	29.21	15.74	26.49	28.56	0.0488	-0.2893	3580	-0.0445	-0.3233
Trachinotus carolinus	NC_024184	16544	28.68	16.27	26.00	29.06	0.0490	-0.2822	3646	-0.0429	-0.3130
Trachinotus ovatus	NC_022707	16564	29.01	15.88	26.23	28.88	0.0490	-0.2903	3564	-0.0399	-0.3246

Trachurus japonicus	NC_002813	16559	27.74	16.58	25.79	29.89	0.0364	-0.2864	3590	-0.0465	-0.3293
Trachurus trachurus	NC_006818	16559	27.71	16.62	25.76	29.91	0.0364	-0.2856	3594	-0.0487	-0.3271
Uraspis helvola	NC_033402	16555	28.15	16.25	25.84	29.77	0.0427	-0.2937	3647	-0.0518	-0.3281
Uraspis secunda	NC_029488	16554	28.17	16.23	25.81	29.78	0.0437	-0.2945	3667	-0.0514	-0.3280

Table S2. Nucleotide composition and skewness levels calculated for sequenced majority strand of the mitogenomes of *Alectis indicus*，*Decapterus tabl* and *Alepes djedaba*.

**Region(s)/Genes**	**Size(bp)**	**Nucleotides Composition**						**AT-Skew**	**GC-Skew**
		A	T	G	C	A+T	G+C		
				Alectis Indicus					
Whole mitogenome	16553	28.03	25.60	16.21	30.16	53.63	46.37	0.045	-0.301
PCGs	11428	25.45	27.69	15.53	31.34	53.13	46.87	-0.042	-0.337
1st condon positions	3800	26.10	20.30	25.50	28.10	46.40	53.60	0.125	-0.048
2nd condon positions	3800	18.30	40.50	13.60	27.70	58.80	41.30	-0.378	-0.341
3rd condon	3800	31.90	22.10	7.60	38.40	54.00	46.00	0.181	-0.670
positions	1556	27.96	26.48	23.84	21.72	54.43	45.57	0.027	0.046
tRNAs									
rRNAs	2667	31.65	20.88	20.92	26.55	52.53	47.47	0.205	-0.119
Control region	861	32.06	30.66	14.63	22.65	62.72	37.28	0.022	-0.215
				Decapterus Tabl					
Whole mitogenome	16545	27.28	25.02	17.05	30.65	52.30	47.70	0.043	-0.285
PCGs	11425	24.33	26.77	16.51	32.39	51.10	48.90	-0.047	-0.324
1st condon positions	3799	25.50	19.70	26.20	28.70	45.20	54.80	0.128	-0.045
2nd condon positions	3799	18.20	40.50	13.60	27.70	58.70	41.30	-0.380	-0.341
3rd condon positions	3799	29.10	20.00	9.90	41.00	49.10	50.90	0.185	-0.611
tRNAs	1555	27.78	26.75	23.99	21.48	54.53	45.47	0.018	0.055
rRNAs	2673	31.72	21.29	21.17	25.81	53.01	46.99	0.197	-0.099

Control region	848	32.55	30.31	15.33	21.82	62.85	37.15	0.036	-0.175
				Alepes Djedaba					
Whole mitogenome	16563	27.92	26.40	16.66	29.03	54.31	45.69	0.028	-0.271
PCGs	11427	25.12	28.59	16.03	30.25	53.71	46.29	-0.064	-0.307
1st condon positions	3798	25.80	21.00	25.90	27.30	46.80	53.20	0.103	-0.026
2nd condon positions	3798	18.20	40.50	13.70	27.60	58.70	41.30	-0.380	-0.337
3rd condon positions	3798	31.10	24.20	8.60	36.10	55.30	44.70	0.124	-0.615
tRNAs	1552	27.90	26.68	23.52	21.91	54.57	45.43	0.022	0.035
rRNAs	2680	31.68	22.09	21.23	25.00	53.77	46.23	0.178	-0.082
Control region	857	33.72	30.11	15.17	21.00	63.83	36.17	0.057	-0.161

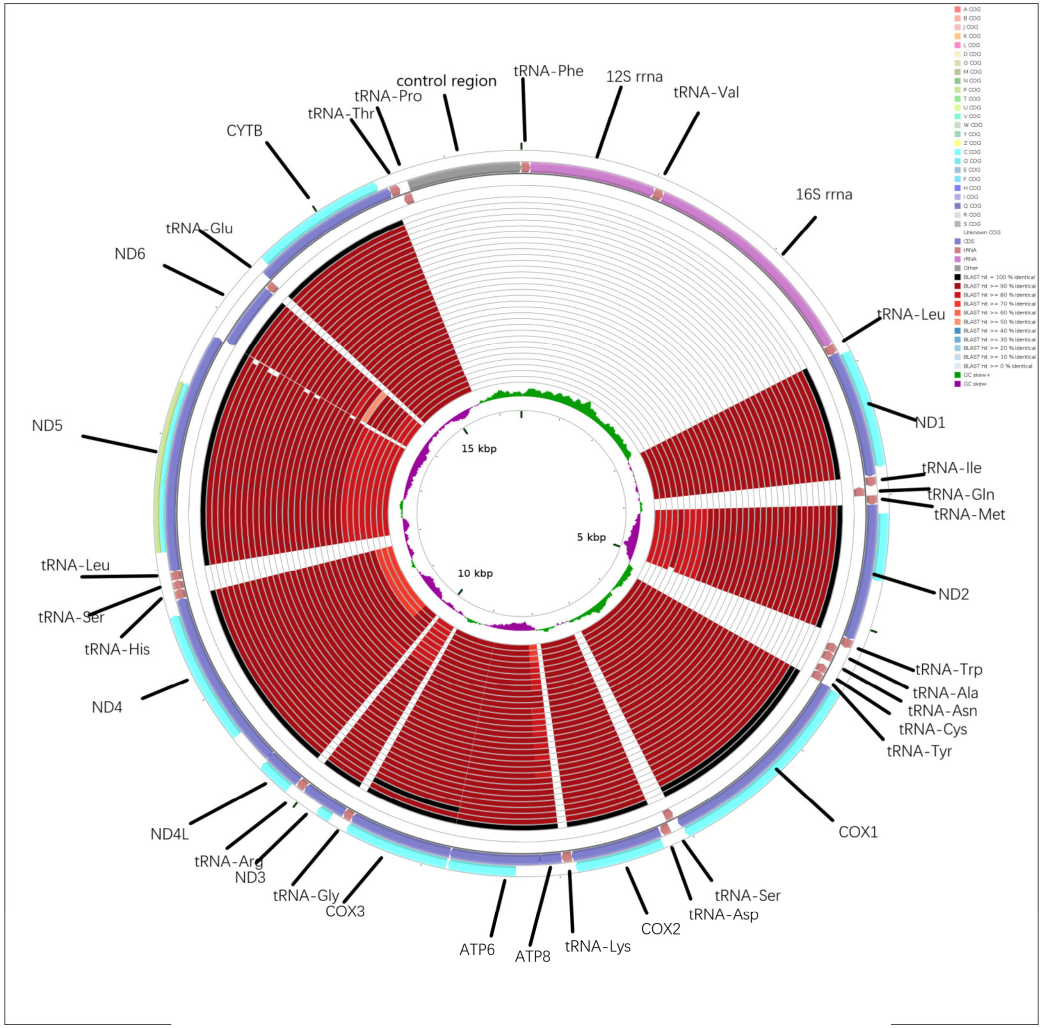

Figure S1. Graphical map of the blast results showing the mitochondrial coding DNA sequence (CDS) identity between *A. indicus* and 31 other Carangidae. Clusters of Orthologous Groups of proteins (COG), gene region, blast identity, and AT-skew are shown from outside to inside. The species from outside to inside are as in Figure 7.

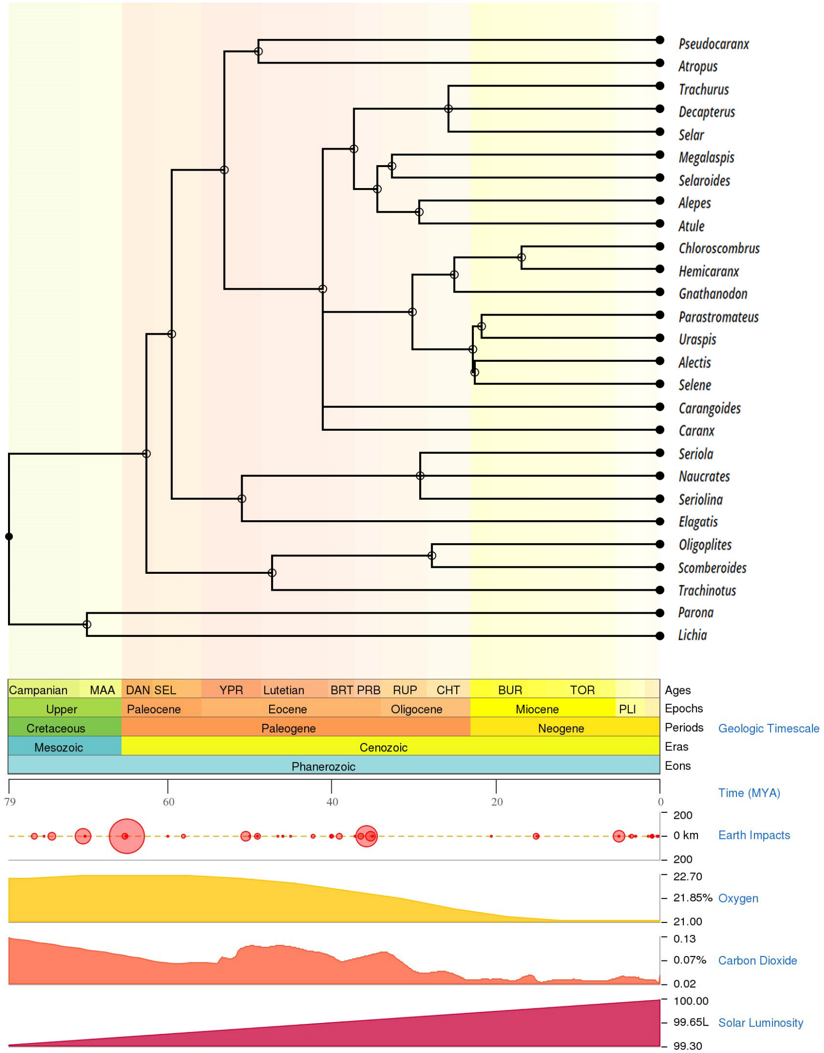

Figure S2. The divergence times of 27 genera of Carangidae generated by the TimeTree database. Changes in environmental parameters during the differentiation of the Carangidae, including earth impacts, changes in the oxygen and carbon dioxide contents, and solar luminosity, are also shown.
